# Developing a non-destructive metabarcoding protocol for detection of pest insects in bulk trap catches

**DOI:** 10.1038/s41598-021-85855-6

**Published:** 2021-04-12

**Authors:** Jana Batovska, Alexander M. Piper, Isabel Valenzuela, John Paul Cunningham, Mark J. Blacket

**Affiliations:** 1 Agriculture Victoria, AgriBio, Centre for AgriBioscience, 5 Ring Road, Bundoora, VIC 3083 Australia; 2 School of Applied Systems Biology, La Trobe University, Bundoora, VIC 3086 Australia

**Keywords:** Bioinformatics, Invasive species, Entomology

## Abstract

Metabarcoding has the potential to revolutionise insect surveillance by providing high-throughput and cost-effective species identification of all specimens within mixed trap catches. Nevertheless, incorporation of metabarcoding into insect diagnostic laboratories will first require the development and evaluation of protocols that adhere to the specialised regulatory requirements of invasive species surveillance. In this study, we develop a multi-locus non-destructive metabarcoding protocol that allows sensitive detection of agricultural pests, and subsequent confirmation using traditional diagnostic techniques. We validate this protocol for the detection of tomato potato psyllid (*Bactericera cockerelli*) and Russian wheat aphid (*Diuraphis noxia*) within mock communities and field survey traps. We find that metabarcoding can reliably detect target insects within mixed community samples, including specimens that morphological identification did not initially detect, but sensitivity appears inversely related to community size and is impacted by primer biases, target loci, and sample indexing strategy. While our multi-locus approach allowed independent validation of target detection, lack of reference sequences for 18S and 12S restricted its usefulness for estimating diversity in field samples. The non-destructive DNA extraction proved invaluable for resolving inconsistencies between morphological and metabarcoding identification results, and post-extraction specimens were suitable for both morphological re-examination and DNA re-extraction for confirmatory barcoding.

## Introduction

Within the last decade, DNA metabarcoding has revolutionised the way biological diversity is measured on a large scale^1–3^. By enabling the simultaneous identification of multiple species within large mixed communities, metabarcoding offers a dramatic reduction in costs compared to traditional morphological identification. Metabarcoding can be used to provide insights into biodiversity in both aquatic and terrestrial environments or obtain high-confidence detection of a small number of species within a background of non-target taxa. A diagnostic approach to metabarcoding is being applied in the monitoring of endangered species^4^, forensic species or food product authentication^5,6^, and invasive species surveillance^7–9^, where assays must also integrate into complex regulatory frameworks. The sensitivity of high-throughput sequencing (HTS) assays are advantageous for these applications, with metabarcoding providing equivalent or better detection than traditional morphological methods and identifying a much wider spectrum of taxa^10,11^. For invasive species surveillance, metabarcoding can detect target species and also previously unrecorded introduced species that have been missed by other approaches^8,12^. Nevertheless, for use in invasive insect surveillance, ensuring the accuracy of detections is paramount as erroneous detections of pest species can lead to severe environmental and economic consequences.

Like all surveillance tools, metabarcoding analyses can be prone to false negative detections when species are missed either due to insufficient sampling as well as biological or technical limitations that cause variations in detectability. Detection errors in metabarcoding can be caused by choice of primers and barcoding loci. The majority of invertebrate metabarcoding studies to date have used the *Cytochrome oxidase I* (COI) gene as their target locus due to the expansive DNA barcode reference library available resulting from a long history of use for molecular species identification^13,14^. However, well-documented issues with PCR bias can result in taxonomic dropout during amplification^15–17^. This bias is thought to primarily arise from primer-template mismatch, and the lack of highly conserved regions within COI to position universal primers has led to other more conserved loci, including 12S, 18S, 16S, Cytochrome b, and NADH, being proposed as alternative targets for metabarcoding^18,19^.

The primary aim of invasive species surveillance is to accurately determine the presence or absence of a pest species^20^; however, obtaining an estimation of population size can also inform management or eradication strategies^21,22^. While there is often a positive correlation between species biomass and the number of sequencing reads^23^, this can be skewed by PCR biases, copy number variation, species richness, and specimen biomass^24–26^. Additionally, the availability and quality of reference sequences needed to identify species can differ greatly between markers and taxonomic groups^8,27^, and sometimes these will need to be generated prior to commencing metabarcoding.

Multi-amplicon and multi-locus approaches can broaden species detection, improve estimates of species abundance, and enable more confident diagnostic metabarcoding by providing independent observations of taxon detections^4,28–30^. Multiplex PCR offers speed and convenience over tandem PCR reactions, making it appealing for high-throughput surveillance applications. The reliability of metabarcoding detections can be confounded by index switching, which occurs when sample-specific indexes recombine, leading to incorrect assignment of sequences^31^. Index switching is reduced by using unique index combinations for each sample and any remaining cross-contaminant sequences can be addressed by a detection threshold^31,32^. Erroneous taxon identification in metabarcoding assays can also be caused by misidentified specimens in reference databases^8,33,34^. While curation of reference databases can overcome this, it can be challenging where reference sequences are sparse or taxonomic synonyms are common^35^. Therefore, detecting species using multiple reference loci produced from a variety of specimens can help strengthen the assay.

Even when primers have been designed around specific target species, metabarcoding can detect non-target taxa^29,36^, which can be problematic for biosecurity surveillance^8,37,38^. A roadblock to using metabarcoding for biosecurity is the inability to validate detections using intact specimens due to the traditionally destructive nature of DNA extraction. Recent metabarcoding studies of bulk invertebrate samples have employed non-destructive methods where DNA is extracted from either the extraction buffer that the specimens have soaked in^39–41^ or the ethanol used to store them^42,43^. The results of these studies suggest that while some taxonomic groups are poorly detected^43^, for arthropods the method is comparable to homogenisation-based approaches^39,41^. Therefore, a non-destructive extraction process could be applied to the bulk insect trap catches collected as part of invasive insect surveillance, to ensure intact specimens remain for confirmation^44^.

In this study we develop a multi-locus, non-destructive metabarcoding protocol for the detection of low-abundance pest insects in bulk trap catches. As a case study, two pest species from the order Hemiptera were chosen; *Bactericera cockerelli* (Šulc) (tomato potato psyllid, TPP) and *Diuraphis noxia* (Mordvilko) (Russian wheat aphid, RWA). TPP is a phloem-feeding insect and important vector of zebra chip disease (*Candidatus* Liberibacter solanacearum) that has recently become established near Perth, Western Australia and is currently restricted to that state through quarantine measures^45^. On the other hand, RWA has recently established in the eastern states of Australia, and following initial detection, was found to be widespread and ineradicable^46^. Both insects have been targets of recent large-scale biosecurity surveillance programs involving costly and labour-intensive morphological processing of hundreds of trap samples^47,48^, and therefore offer a suitable model system for evaluating metabarcoding in a diagnostic context.

## Materials and methods

### Insect rearing and field trapping

To assemble mock communities of known composition, colonies of psyllids (*Acizzia solanicola* Kent & Taylor and *Acizzia alternata* Kent & Taylor) and aphids (*Rhopalosiphum padi* L., *Metopolophium dirhodum* (Walker), and RWA) were established using field-collected individuals from a range of hosts and localities in Victoria, Western Australia, and New South Wales, Australia (Table 1). The colonies were reared on eggplants (psyllids) or barley (aphids) in a controlled environment room at 20 °C ± 2 and 65% ± 5 RH in BugDorm-4F3074 insect cages (MegaView Science Co.). After 4–6 weeks of colony development, adult specimens were collected and stored in absolute ethanol at − 20 °C. In addition, ethanol-preserved specimens of TPP were provided by DPIRD, Western Australia. All of the psyllid and aphid adult specimens used were similar in size. Taxonomic keys were used to morphologically confirm the identity of aphids^49^, psyllids^50^, and TPP^51^. Voucher specimens were deposited in the Victorian Agricultural Insect Collection (VAIC) and associated tissue collection (VAITC) held at the AgriBio Centre, Bundoora, Australia (Table 1). Mock communities with total abundances of 100, 250, 500, and 1000 individuals (n = 5 of each size) and varying species composition were assembled (Table S1). The number of species per mock community ranged from three to six, with approximately similar proportions across the four sizes. Differing numbers of TPP and RWA specimens were included in the pools in order to determine assay sensitivity. The word ‘pool’ is used to refer to each mock community sample. The 100 and 250 pools were stored in 2 mL tubes, whereas the 500 and 1000 pools were stored in 50 mL tubes.

**Table 1:** Aphid and psyllid species used in the mock communities and associated collection details.

Insect species	Collection date	Locality	Host	VAIC/VAITC
*Acizzia solanicola* Kent & Taylor	January 2015	Brunswick East, Victoria	*Solanum mauritianum*	7086
*Acizzia alternata* Kent & Taylor	April 2015	Bellingen, New South Wales	*Solanum mauritianum*	7087
*Bactericera cockerelli* (Šulc)	August 2017	Bunnings Perth, Western Australia	*Capsicum annuum*	6910
*Rhopalosiphum padi* (L.)	June 2009	Horsham, Victoria	*Avena sativa*	7088
*Metopolophium dirhodum* (Walker)	March 2017	Yea, Victoria	Poaceae	7091
*Diuraphis noxia* (Mordvilko)	June 2016	Horsham, Victoria	Wild oats	7090

*VAIC* Victorian Agricultural Insect Collection, *VAITC* Victorian Agricultural Insect Tissue Collection.

In addition to rearing aphid and psyllid colonies for the mock communities, field surveys were conducted in order to acquire samples representative of biologically relevant diversity. Four Macquarie Island traps^52^ were deployed at Blampied, Victoria bordering organic potato and vegetable crops for 6 weeks from December 2017 to January 2018. These traps collected windborne insects into a collection vial containing 300 mL of 50% propylene glycol and a small quantity of borax. Propylene glycol has been shown to preserve both morphological features and DNA quality^53^. Trapped specimens were collected weekly, and upon arrival to the laboratory sorted by size using a stereo microscope. Specimens greater than 0.5 cm were removed and the remaining specimens transferred into absolute ethanol in a 2 mL tube. Ten of the best-preserved trap samples containing the greatest numbers of specimens were selected for metabarcoding analyses. The proportion of Hemiptera within each trap sample was determined via morphological examination, photographed, and returned to the sample. The trap samples were further examined in detail to determine the presence of RWA or TPP. Once absence of TPP was confirmed, ethanol-preserved TPP specimens were used to spike three of the trap samples prior to DNA extraction (Table 2)*.*

**Table 2:** Number of specimens from field survey traps used for metabarcoding analyses, with a comparison of the percentage identified as Hemiptera by morphology and metabarcoding. The metabarcoding identification is based on sequencing reads from all three loci. Bold highlighting indicates traps where metabarcoding unexpectedly detected pest species, with the proportion of sequencing reads attributed to RWA or TPP in brackets.

Trap number	Total insect specimens	% Hemiptera (morphology)	% Hemiptera (metabarcoding)	RWA^a^	TPP^b^
Trap 1	180	38.3	87.6	3	1
Trap 2	192	18.2	49.5	**0 (0.14%)**	0
Trap 3	224	44.4	50.3	**0 (0.11%)**	0
Trap 4	86	47.7	86.4	3	2
Trap 5	111	20.7	25.2	1	0
Trap 6	118	25.4	27.4	3	0
Trap 7	56	37.5	88.9	8	3
Trap 8	140	41.4	40.2	27	**0 (0.04%)**
Trap 9	40	32.5	32.3	4	0
Trap 10	121	43.0	84.3	15	**0 (0.03%)**

^a^Russian wheat aphid (RWA) specimens from field surveys.

^b^Tomato potato psyllid (TPP) added from ethanol-preserved specimens from Western Australia.

### DNA extraction

**Figure 1:**
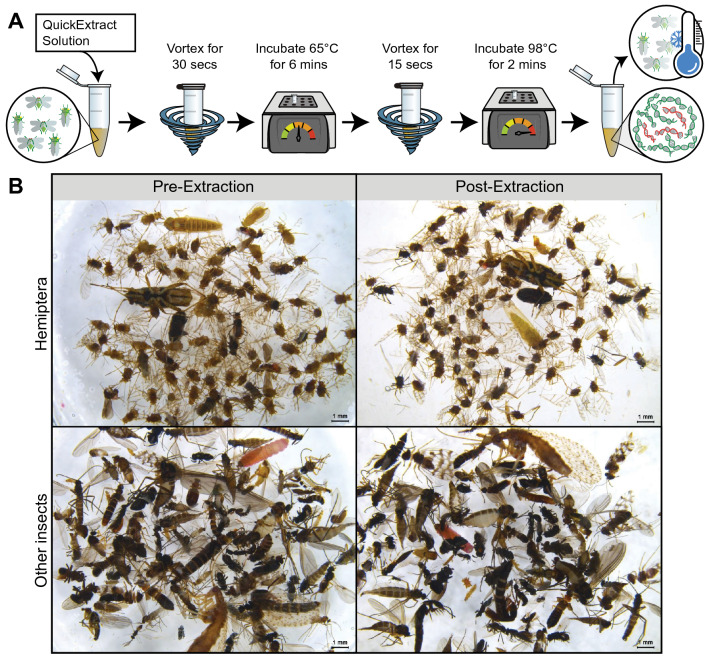
(**A**) Non-destructive DNA extraction protocol newly developed in this study. (**B**) Specimens from a trap sample before (left) and after (right) non-destructive DNA extraction using QuickExtract. Both Hemiptera (above) and other insect (below) specimens are preserved.

DNA was extracted from both the mock communities and trap samples using a newly developed non-destructive method (Fig. 1). Firstly, ethanol was removed from the insect pools using a 1000 μL pipette and the specimens were air-dried in the tubes for 10 min. 250 μL of QuickExtract DNA Extraction Solution (Lucigen) was added per 100 specimens, ensuring all insects were immersed in the buffer. Specimen tubes were vortexed at 1400 RPM for 30 s, followed by a 6 min incubation at 65 °C, vortexed for 15 s, followed by a 2 min incubation at 98 °C (as per the manufacturers protocol). Approximately 250 μL of QuickExtract solution containing extracted DNA was then transferred to a new 1.5 mL tube, quantified using a NanoDrop 1000 spectrophotometer (Thermo Fisher, USA), normalised to 40 ng/μL, and stored at − 20 °C until PCR amplification. To assess morphological preservation of specimens, the trap samples were re-examined to determine the proportion of Hemiptera, photographed, and returned to the sample. All insect pools were resuspended in 1–20 mL of absolute ethanol and returned to storage at − 20 °C.

### Primer design

In order to design appropriate metabarcoding primers for a multiplexed assay, reference sequences for COI, 12S, and 18S loci were obtained from all the species from the live insect colonies and TPP from WA (Table 1) using both published^54–56^ and novel primers (Table S2). All three loci successfully differentiated all species, except for *A. alternata* and *A. solanicola* which could not be differentiated by 18S and therefore have been aggregated throughout the metabarcoding analysis. The generated reference sequences (GenBank accessions MW804274-MW804279; MW804905-MW804916) were aligned in Geneious R8.1^57^ and novel metabarcoding primers for each locus were selected (Table S3). The amplicon size for these loci ranged from 337 to 398 bp, which included the Illumina adapter sequences attached to the 5′-end of both forward and reverse primers. Where possible, nucleotide variation between target species was accounted for by placing degenerate bases towards the 3′-end of the primers.

### Amplicon library generation

The amplicon libraries were prepared in three batches: (1) replicated sets of the 5 × 250 mock communities to compare combinatorial and unique dual indexing strategies; (2) the 5 × 100, 5 × 500, and 5 × 1000 mock communities; (3) the 10 field trap samples. Amplicons were generated using multiplex PCR in which all three target genes were amplified in a single reaction per sample using the three sets of metabarcoding primers. Each 25 µL reaction consisted of 5 μL of 5 × MyFi reaction buffer (Bioline), 15 nM each SternoCOI_F and SternoCOI_R primers, 10 nM Sterno12S_F and Sterno12S_R primers, 2.5 nM Sterno18S_F and Sterno18S_R primers, 0.8 μL MyFi DNA polymerase (Bioline), 11.2 μL BSA (NEB), and 2.5 μL of 40 ng/µL template DNA. Cycling conditions were 94 °C for 2 min, 30 cycles of 94 °C for 30 s, 50 °C for 45 s, and 72 °C for 45 s, followed by a final extension step of 2 min at 72 °C. The multiplexed amplicons (COI, 12S, and 18S loci) were then verified on 2% w/v agarose gels and purified using a 0.8:1 ratio of Agencourt AMPure XP beads (Beckman Coulter).

Real-time PCR was used to attach 8 bp sample indexes and sequencing adapters to each of the amplicons. To investigate the impacts of index switching on the sequencing data, the 250 mock community libraries (pools 1–5) were prepared using both non-unique (combinatorial) and unique dual indexes. The remaining three sets of 5 mock communities (100, 500, and 1000) as well as field trap samples were prepared using unique dual indexes. The indexed amplicon libraries were purified using a 0.8:1 ratio of AMPure XP beads, quantified using a 2200 TapeStation (Agilent Technologies) with the D1000 ScreenTape assay, and pooled in equimolar ratios. A Qubit 3.0 Fluorometer (Life Technologies) was used to quantify the pooled libraries, which were then diluted to 7 pM with a 15% PhiX spike-in. The three batches of amplicon libraries were sequenced across three flow cells on the Illumina MiSeq platform (2 × 250 bp reads).

### Reference database assembly

To assemble training datasets for taxonomic classification by the Ribosomal Database Project (RDP) naïve Bayesian classifier^58^, all COI, 12S, and 18S arthropod sequences were retrieved from the Barcode of Life Data System (BOLD)^59^ and NCBI GenBank database^60^ using the bold v0.5.0^61^ and rentrez v1.2.1^62^ packages in R v3.4.4^63^. Hierarchical taxonomy for each sequence was retrieved from specimen records (BOLD sequences) or through the GenBank Taxonomy Database using the taxonomizr v0.5.2^64^ R package, and only taxa with complete binomial species names retained. Sequences for each locus were then mapped against their respective set of reference sequences used to design primers using the mapper at medium sensitivity in Geneious R11.1^57^ and then trimmed to primer binding regions.

**Figure 2:**
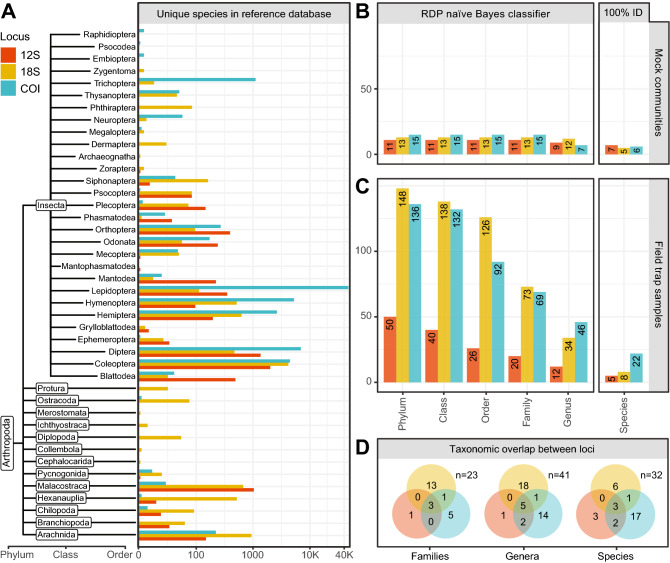
(**A**) Taxonomic composition of the reference database, displaying number of unique species for each order within Insecta, as well as for other classes of Arthropods. Number of amplicon sequence variants (ASVs) from (**B**) mock communities and (**C**) field trap samples successfully assigned to taxonomic ranks for each locus using the Ribosomal Database Project (RDP) naïve Bayesian classifier and exact matching with reference sequences. (**D**) Taxonomic overlap between loci at the family, genus, and species level for all samples.

Erroneous or taxonomically mislabelled public sequences were filtered out by removing: (1) duplicate sequences and those larger than 3000 bases or smaller than 200 bases; (2) records containing terms indicating insufficient identification (i.e. sp., nr., aff.—see Appendix S1 for full list of terms); (3) sequences matching a local database of *Wolbachia* sequences with > 95% identity using a BLASTn v2.7.1 search; (4) misannotated sequences as determined by BLASTn searching sequences from clusters with multiple associated phyla, classes or orders (99% similarity clustering performed using SUMACLUST v1.0.31^65^). Post-filtering, datasets for each locus were merged together with the in-house sequences used to design the metabarcoding primers and formatted as required for the RDP classifier and exact matching functions implemented in the DADA2 R package^66^. The taxonomic composition of these final training sets can be seen in Fig. 2A and have been uploaded to 10.5281/zenodo.3557020.

### Bioinformatics analysis

Demultiplexed MiSeq reads (NCBI SRA acc no: PRJNA716058) were trimmed of PCR primers and sequencing adapters using BBDuK in BBTools v38.01^67^. Sequence quality profiles were used to filter reads with more than two expected errors or ambiguous ‘N’ bases and all remaining sequences > 100 bp were then analysed using DADA2 v1.9.3^66^. As error rates can vary between flow cells and libraries, the DADA2 error model was determined separately for each MiSeq flow cell and visualised to ensure correct fit before reads were denoised. Following denoising, the inferred amplicon sequence variants (ASVs) from each MiSeq flow cell were combined into a single table, and chimeras were detected and removed de-novo using the *removeBimeraDenovo* function in DADA2 (available at 10.5281/zenodo.3557020). Taxonomy was assigned to the 2152 ASVs to the lowest rank possible with a minimum bootstrap support of 80% using the RDP classifier as implemented in the DADA2 R package, followed by species level assignment using exact matching between the query and reference sequences. As the taxonomic training sets only covered Arthropoda, all sequences that could not be reliably assigned to this phylum were excluded from analysis, and COI sequences were aligned using MACSE v2.01^68^ to further identify and remove any sequences containing frame shifts and stop codons that commonly indicate pseudogenes. While rRNA pseudogenes that may affect 12S or 18S loci can also occur, their identification is more challenging^69^ and therefore this was only conducted for COI ASVs.

To determine the overall rate of index switching, MiSeq data from the 100, 500, and 1000 pools were demultiplexed using bcl2fastq Conversion Software v2.20 (Illumina) and the indexes were summarised from FASTQ headers for both the determined and undetermined reads. These indexes were compared to a list of all possible combinations of i5 and i7 indexes and the overall contamination rate (1.08%; Figure S1) was calculated from the ratio of valid (applied during library preparation) to invalid (pairs that could only arise due to switching) combinations^70^. As this contamination rate represents switching at either the i5 or i7 index, the square of this (0.01%) approximates the residual misidentification rate that could occur through switching of indexes at both ends of the molecule^32^. Any taxa with a per-library relative abundance below this threshold were filtered in R. Community level metrics for the final species occurrence table (available at 10.5281/zenodo.3557020) were visualised using phyloseq v1.22.3^71^ and ggplot2 v3.1.0^72^.

### Confirmation of metabarcoding results

Any unexpected or potential exotic detections (i.e., first records for either the state of Victoria or Australia) revealed by the metabarcoding analysis were confirmed using the preserved insects that were stored following non-destructive DNA extraction. The samples were morphologically inspected for any flagged species and DNA was re-extracted from individual specimens using a 5% Chelex 100 resin (BioRad) method following Walsh et al.^73^. Briefly, aphids were placed in 1.5 mL tubes containing 5 µL of Proteinase K (Qiagen) with 2 glass beads and crushed in a mixer mill for 1 min at 30 Hz. Then 150 µL of 5% Chelex was added and the extract incubated at 55 °C for 3 h, then at 85 °C for 8 min, and stored at − 20 °C. A PCR was performed to amplify COI from these specimens using the SternoCOI_F and SternoCOI_R primers (Table S3, excluding the adapter sequences). The 25 µL reactions consisted of: 14.8 µL of 1 × BSA, 2.5 µL of 10 × ThermoPol Reaction Buffer, 2 µL of 2.5 μM dNTPs, 1.25 µL of 10 μM each of SternoCOI_F and SternoCOI_R primers, 0.2 μL of MyTaq™ DNA polymerase (all Bioline), and 3 μL of template DNA. Cycling conditions were the same as those used for amplicon library generation. PCR products were sequenced by Macrogen Inc. (Seoul, Korea) and sequences were compared to public databases using BOLD and NCBI databases to identify specimens. MEGA X^74^ was also used to create an alignment (233 bp) of the sequences with ClustalW and build a neighbour-joining tree using p-distances and a bootstrap analysis with 1000 replicates.

## Results

### Performance of metabarcoding using mock communities

**Figure 3:**
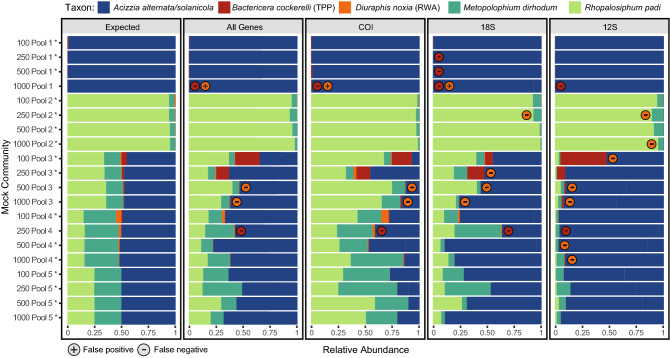
The expected (based on number of individuals) and observed (based on sequencing reads) relative abundance of each species in each mock community pool. *Acizzia alternata* and *A. solanicola* are aggregated for display purposes as these species could not be differentiated by the 18S loci. Observed relative abundance data is shown for the mean across the three loci, and for COI, 18S, and 12S separately. False positive and negative genera are indicated in each pool based on a detection threshold of 0.01%. Asterisks (*) indicate pools that had all species correctly identified.

**Figure 4:**
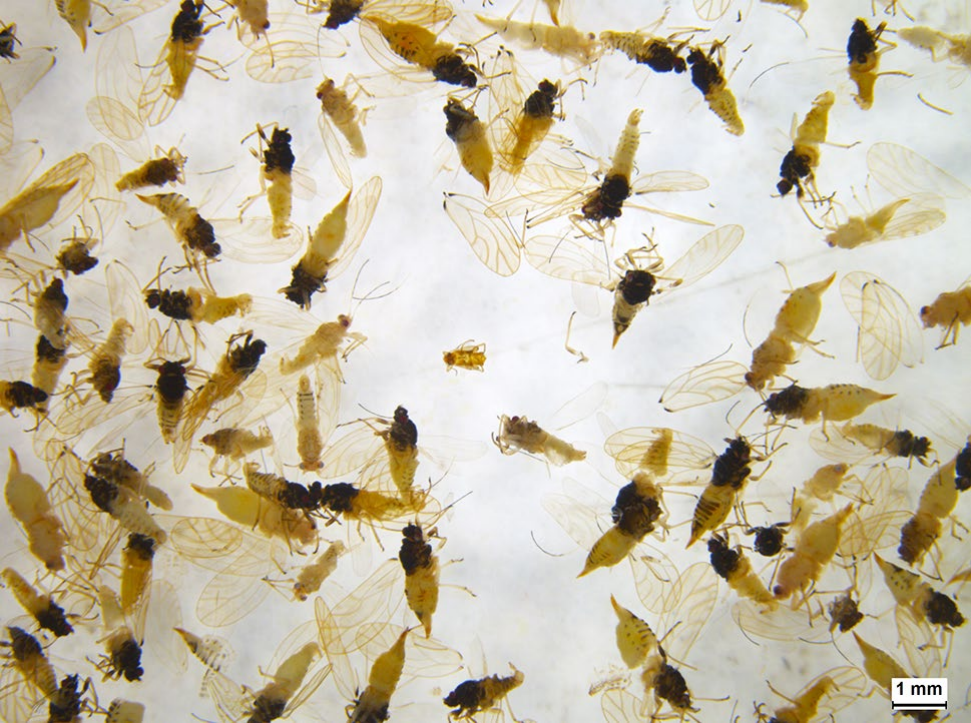
The *Diuraphis noxia* (Russian wheat aphid) nymph specimen responsible for the apparent “false positive” reads detected in the 1000 Pool 1 mock community, following non-destructive DNA extraction.

When using data from all three loci, metabarcoding successfully detected all species present in 80% of the mock community pools (Fig. 3 and Table S4). There were no false positive or negative detections in Pools 2 or 5, regardless of size. This contrasted with results in Pools 1, 3, and 4 which contained false negatives, where metabarcoding failed to detect TPP and RWA in some larger pool sizes (a single TPP specimen in 1000 Pool 1 and 250 Pool 4, and also a single RWA specimen in 500 Pool 3 and 1000 Pool 3). Interestingly, the assay detected RWA at a relative abundance of 0.13% in 1000 Pool 1, in which this species was thought to be absent. Re-examination of the preserved specimens from 1000 Pool 1 revealed the presence of an aphid nymph that was accidently placed in this pool (Fig. 4). A new DNA extraction (destructive) was performed on this specimen and it was identified as RWA via COI barcoding (SternoCOI_F and SternoCOI_R primers), therefore confirming the source of the RWA reads in 1000 Pool 1.

Observed abundance (based on sequencing reads) for each species generally reflected the expected abundance (based on number of individuals). However, biases were seen in certain species across all four pool sizes, resulting in higher or lower observed abundances compared to expected (Fig. 3). In particular, relative abundances of *A. alternata/solanicola* were generally greater than expected while abundances of *M. dirhodum* were lower than expected (Fig. 3).

Index switching was significantly reduced when unique dual indexes were used compared to combinatorial indexes. When combinatorial indexing was used for the five 250 mock community pools, a total of 3624 false positive reads from taxa known to be absent were detected across all communities. In contrast, when unique dual indexing was used for the same samples, only 53 false positive reads were observed, greatly improving the limits of detection. When looking at the number of reads assigned to invalid combinations (i.e. those filtered out by use of unique dual indexes), the rate of index switching did not show a relationship with the edit distance between index combinations (Figure S2), suggesting the cause of this switching was not sequencing error within the index reads.

### Field survey traps

**Figure 5:**
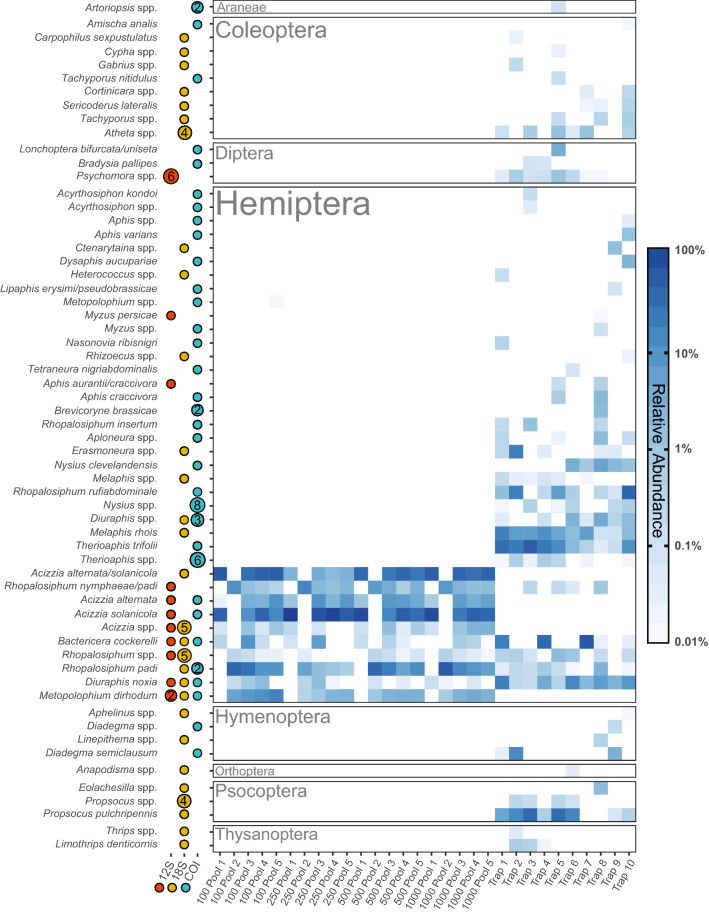
Heat map displaying the relative abundance of different Arthropoda taxa in both mock community and field trapped insect samples based on sequencing reads. Taxa designated ‘spp.’ denote genus-level classification. The loci contributing to detection of each taxa is indicated by a red (12S), yellow (18S), or blue (COI) dot. The size and number inside the dot indicate if more than one amplicon sequence variant (ASV) was assigned to the taxonomic rank. Mean relative abundances across all three amplicons are displayed on a log10 scale. Only ASVs that could be reliably classified to genus with a relative abundance above 0.01% are reported.

Morphological analysis determined RWA was present in all the field trap samples except for Traps 2 and 3 (Table 2). However, metabarcoding detected RWA in every trap sample (Fig. 5). Upon re-examination of the preserved specimens of the trap samples, an adult RWA was found in Trap 3 which was missed by the initial assessment of the traps, and also an unidentified aphid nymph in Trap 2. Despite a follow-up DNA extraction (destructive) of the nymph, PCR amplification from this specimen was not successful, however this could explain the RWA reads in Trap 2.

For TPP detection, preserved specimens from Western Australia were used to spike Traps 1, 4, and 7 (Table 2). However, TPP was also detected in Traps 8 and 10 (Fig. 5). Re-examination of Traps 8 and 10 did not reveal any TPP specimens. Examination of the metabarcoding reads revealed a large bias towards TPP in the three spiked traps, with up to 84% of reads from TPP. Only 0.04% of Trap 8 and 0.02% of Trap 10 reads were attributable to TPP.

In addition to RWA and TPP, metabarcoding detected a wide variety of non-target arthropod species within the field trap samples. Compared to the 28 ASVs assigned to genus in the mock communities (Fig. 2B), there were 92 ASVs assigned to genus in the field trap samples (Fig. 2C). When taxonomic overlap between the three loci was considered, 23 unique families, 41 unique genera, and 32 unique species were detected across the whole study (Fig. 2D). In a number of cases, multiple unique ASVs arising from the same loci mapped to a single genus or species and could represent multiple taxa or intraspecific diversity. This can also arise from errors introduced during early PCR cycles. The proportion of Hemiptera in the traps ranged from 25.2–88.9% based on ASVs, compared to 18.2–47.7% as determined by morphological identification (Table 2).

Three trapped species tentatively detected by the metabarcoding assay have not previously been recorded in Australia: *Melaphis rhois*, *Carpophilus sexpustulatus,* and *Aphis varians*^75^. Aphid species *M. rhois* was only detected by 18S at varying abundances across all trap samples. However, despite being an exact match, the 18S reference was the only representative sequence for the *Melaphis* genus. Similarly, the sap beetle species *C. sexpustulatus* was detected by 18S in Trap 2 with an exact match to a reference sequence. However, further exploration using a BLASTn search against the NCBI GenBank database revealed sap beetles have very low interspecific distances for the 18S region used, resulting in poor species level resolution. Due to the conserved nature of 18S and paucity of reference sequences it is highly likely that both tentative detections represent endemic species for which 18S reference data does not yet exist. In contrast, the aphid species *A. varians* was detected by COI (1611 reads) in Trap 10 with exact matches to several reference sequences, and therefore is more likely to represent a real detection. The preserved specimens from Trap 10 were checked for the presence of *A. varians* and parts of unidentifiable aphids were found (e.g. heads and abdomens) as well as a number of aphid instars. Destructive DNA extractions were performed on 10 aphid parts and nymphs, with one abdomen sample producing a 100% match based on the COI barcode (SternoCOI_F and SternoCOI_R primers) to *A. varians* reference sequences in BOLD (Figure S3). The remaining samples did not result in successful amplification.

### Comparison between loci

When each of the three loci were analysed separately using the mock community data, 12S, and 18S resulted in more false negative and positive results than COI (Fig. 3). Analysis of COI data resulted in four false negatives and one false positive, which is identical to when data from all three loci were combined. Conversely, using only 12S resulted in nine false negatives, and 18S resulted in eight false negatives and one false positive. This suggests that using solely COI as a metabarcoding marker would result in similar species recovery from a pooled sample as would using COI, 12S, and 18S combined. However, the use of 12S and 18S helped to produce more accurate abundance estimates for the *A. alternata/solanicola*, *M. dirhodum,* and *R. padi* species in the combined loci analysis by countering the bias produced by COI for these species (Fig. 3).

The number of sequencing reads attributed to each locus was affected by taxonomic composition of the sample (Table S5). When only psyllids were present, there was a strong bias against COI, with only 7.96–23.87% of the total reads attributable to COI. However, when only aphids were present in the pool, there was a strong bias towards COI (58.63–87.07%) and a strong bias against 12S (4.04–10.31%). The number of reads attributed to loci also showed signs of batch effects between experiments, which can largely be attributed to the PCR performed. The 250 mock community pools were amplified separately to the other pools and had poor amplification of 18S (Figure S4). This resulted in a low number of 18S sequencing reads for the 250 mock community pools (1.7–4%) compared to the other pools (27–56%).

Of the 32 unique species detected in both the mock communities and the field traps, only TPP, RWA, and *M. dirhodum* (9%) were successfully detected with all three loci, owing to the prior generation of in-house reference data for these species (Fig. 2C; Fig. 5). *Rhopalosiphum padi* was detected with COI and 18S, however, could not be assigned to species level with 12S because the sequence was identical to several species of *Rhopalosiphum.* Similarly, *A. alternata* and *A. solanicola* (6%) were detected with COI and 12S only, as the 18S amplicon lacked resolution to separate the two. The remainder of the species were detected by a single locus: 17 (53%) by COI, six (19%) by 18S, and three (6%) by 12S. On the other hand, of the 41 unique genera detected, five (12%) were detected with all three loci, one (2%) was detected with both COI and 18S, and two (5%) were detected with both COI and 12S. Fourteen (34%), 18 (44%), and one (2%) genus were detected exclusively with COI, 18S, and 12S respectively (Fig. 2C). In the trap samples, a 12S ASV exactly matched reference sequences of both *R. nymphaeae* and *R. padi*, and another matched both *A. aurantii* and *A. craccivora*. Similarly, a COI ASV exactly matched reference sequences of both *Lonchoptera bifurcata* and *L. uniseta*, and another matched both *Lipaphis erysimi* and *L. pseudobrassicae*. While our reference database underwent a curation process, this could indicate remaining misidentified taxa that were not sufficiently resolved, or issues with the underlying taxonomic delimitation of these species.

## Discussion

In this study we assessed the ability of metabarcoding to detect low abundance pest insects within mock communities of aphid and psyllid species, and then validated the approach on field-trapped insects collected from potato and vegetable crops. Metabarcoding of mock communities indicated that while all species were usually detected when all three loci were used, an increase in the number of individuals in a pool led to a decrease in detection of single specimen species (Fig. 3; Table S1 and S4). The rate of missed detections increased when only 18S or 12S data was used but remained the same with only COI data, which is likely due to COI having a favourable bias profile towards the targets. Inability to recover low frequency taxa is a common finding in metabarcoding studies^11,21,76^ and can be exacerbated by non-destructive DNA extraction methods. For instance, soft-bodied taxa are more likely to be detected with metabarcoding using non-destructive DNA extraction than taxa with greater levels of sclerotization^39^. This may also explain why metabarcoding identified more Hemiptera in the field survey traps than morphological identification methods did (Table 2). Nevertheless, sequencing reads for all of the mock community taxa were present in the raw data but were under the detection threshold required to remove index switching.

We found the use of unique dual indexes dramatically reduced the rate of index switching compared to combinatorial indexing, thereby enabling a lower detection threshold and increasing sensitivity of the metabarcoding assay. However, even with unique dual indexes, low rates of index switching can still be seen due to rare occurrences of switching at both ends of the molecule^32^. The use of the Free Adapter Blocking Reagent has been recommended to further reduce index switching caused by free adapters on Illumina ExAmp chemistry (i.e. HiSeq, NovaSeq)^77^; however, it is unclear how this would affect the bridge amplification-based cluster generation of the Illumina MiSeq platform used in this study. Choosing an appropriate filtering threshold to remove cross-contamination remains a challenge for metabarcoding studies^2^, as our filtering threshold derived from the mock communities did not enable detection of TPP in the field trap samples. Furthermore, while our threshold was based on index switching rate, our approach did not account for well-to-well contamination during library preparation. This has recently been raised as a major source of contamination, especially when libraries are prepared in microtiter plates or in automated liquid handling systems^78^. A more robust method of estimating cross-contamination may be to include a positive spike-in control during DNA extraction in the form of a taxa alien to the target environment^79^ or a synthetic sequence^80^.

While metabarcoding was not able to detect all the species present in the mock communities, it did reveal the presence of a pest insect that was missed by morphological identification. The presence of RWA in the 1000 Pool 1 mock community was initially thought to be a false positive (Fig. 3); however, re-examination of the preserved mock community specimens revealed an RWA nymph (Fig. 4), highlighting the value of non-destructive DNA extraction. COI barcoding of the nymph demonstrated that non-destructive DNA extraction preserves specimens adequately for both morphological identification (Fig. 1) and/or individual barcoding. Metabarcoding also detected RWA in field traps 2 and 3, despite this species not being recorded in the initial morphological identification. Re-examination revealed an aphid nymph in Trap 2 and an RWA adult in Trap 3. However, PCR amplification of the COI barcode was not possible for the nymph specimen, which could be due to field trap specimens having more degraded DNA compared to the mock community specimens. While the COI primers were designed to amplify a relatively short region of COI (337 bp; Table S3), perhaps an even shorter region could help to improve amplification from the field trap specimens, with barcodes as small as 100 bp successfully used for species identification^81^.

Unlike RWA, no specimens were found to confirm the TPP detections in Traps 8 and 10. The erroneous TPP detections could have been caused by cross-contamination during library preparation, and future studies should include negative controls to provide a cumulative measure of physical contamination^82^. While we cannot rule out physical contamination, the strong PCR bias toward TPP could have led to increased index switching^83^. This bias was present in all three loci in the field trap samples and not present in the mock community results (Fig. 3), suggesting that degraded DNA in trapped specimens could be flooded by well-preserved DNA from the spiked TPP specimens. Further study using field-trapped TPP is required to determine the suitability of metabarcoding for surveillance of this pest species.

Quantitative estimations of the three multiplexed loci were impacted by the overall ratio of aphids to psyllids within the pools (Table S5) and PCR batch effects (Figure S4). The impact of PCR batch effects on quantitative estimates was greatest in 250 Pool 3, where the abundance estimates varied considerably for the larger and smaller community size with the same composition (100 Pool 3, 500 Pool 3, 1000 Pool 3) that were run in a different PCR batch (Fig. 3). The large difference may be due to the confounding factors of PCR batch effects, primer biases associated with community composition^26^, and PCR competition between loci. This indicates that tandem rather than multiplexed PCR reactions or microfluidic multiplexing^29^ may be more appropriate for quantitative estimates in multi-locus assays. Furthermore, we suggest future studies include identical mock communities across each library preparation and sequencing run to allow estimation and correction of these batch effects^84,85^. These calibration communities could also be used to derive correction factors to account for taxon-specific quantitative bias^25,86,87^. However, assembling appropriate calibration communities may be difficult for the diverse range of species captured by the wind-based surveillance traps used in this study. Therefore, if accurate abundance estimates are necessary then an approach that does not utilise PCR, such as hybridisation probes/capture baits^70,88,89^ or whole mitochondrial genomes^90^, may help to improve quantitative estimates. Nevertheless, these techniques still possess their own individual biases^86^ and do not currently have wide acceptance in validated diagnostic protocols^8^.

Despite a Hemiptera-based primer design (Table S2), the sequences from the field survey traps revealed a broad diversity of Arthropod species (Fig. 5), including three tentative first detections for Australia. The most notable was the detection of the aphid species *A. varians* in Trap 10, identified to species level through exact matching of COI to reference sequences in public databases and confirmed via COI barcoding of an aphid abdomen found upon re-examination of Trap 10 (Figure S3). *Aphis varians* is a Neartic species that belongs to a complex group associated with wild and cultivated *Ribes* spp. (Grossulariaceae) as primary hosts and *Epilobium* spp. (Onagraceae) as secondary hosts^91^. In Australia, there are no records of aphids causing damage to commercial *Ribes* spp. (currants and gooseberries), including the recently introduced and closely related *A. oenotherae*. Nevertheless, the detection of a new aphid associated with *Ribes* spp. warrants further investigation and surveys from hosts such as *Epilobium* spp. that are common in Australia. Importantly, the detection of *A. varians* from a lone abdomen represents a situation that would not have occurred when following a conventional diagnostic approach, and indeed this taxon was overlooked during the initial sorting of the traps. Laboratories conducting insect diagnostics are unlikely to individually barcode every incomplete specimen in a trap sample due to the significant costs involved, and this demonstrates the effectiveness of a non-destructive metabarcoding approach for flagging samples that contain unexpected non-target taxa, which can then be more thoroughly inspected and confirmed using conventional diagnostic methods.

In contrast to the detection of *A. varians*, the other tentative first detections for Australia, *M. rhois* and *C. sexpustulatus*, were indicated only by the 18S locus. While the *M. rhois* detection was based on an exact match to 18S, this reference sequence was the only representative for its genus. Singletons such as these present problems for taxonomic classification because there is no way to calibrate the assignment confidence with the taxonomy and sequence similarity of closely related sequences^92^, and therefore they are often removed from reference databases^93^. However, in this case, removal of singletons would have resulted in loss of a large proportion of the 18S and 12S references, which already have marginal representation in the database (Fig. 2A). This issue is further compounded by the highly conserved nature of 18S, which while useful for detecting a broad diversity of taxa at higher taxonomic ranks (Fig. 2C), can struggle to differentiate many taxa at the species level^94^. For example, in the case of the *C. sexpustulatus* detection, all reference 18S sequences for this genus showed less than 1% variation, so while this was assigned to species with an exact match, it likely represents a closely related native *Carpophilus* spp. for which an 18S reference sequence does not currently exist^95^. Furthermore, while the RDP classifier used in this study has previously performed well with COI where there is a broad diversity of reference sequences^96,97^, it can suffer from over-classification in the case of sparse reference data^98^. Therefore, for loci other than COI to be effective, a greater emphasis needs to be placed on conducting baseline surveys and improving the taxonomic coverage of reference databases for endemic species at the beginning of a surveillance program.

As DNA metabarcoding begins to be applied in diagnostic situations, increasing regulatory confidence will be critical for widespread uptake. While the multi-locus assay did not perform effectively in providing validation of detections, this was primarily due to insufficient availability of reference sequences for loci other than COI. Due to the already widespread availability of reference databases and high resolution for species-level discrimination, we recommended the use of COI with additional PCR replicates for metabarcoding studies aiming to detect insect pests. On the other hand, non-destructive DNA extraction proved extremely useful for validating detections, enabling confirmation of both target insects and off-target species *A. varians*, which has not been previously recorded in Australia. While in this case *A. varians* is not a serious pest, the ability to detect off-target insects may help prevent situations similar to the initial establishment of RWA in Australia, where a surveillance program was not initiated until after the first detection, revealing an already widespread distribution beyond the hope of eradication^46^. Furthermore, non-destructive DNA extraction could be used to continuously build up local databases over the course of a surveillance program^44^. For example, in this study many of the trap samples contained ASVs that were unable to be assigned to genus or species (Fig. 2C) and these could be revisited to locate and generate reference information for previously unbarcoded taxa. This circular workflow would greatly aid the timely implementation of metabarcoding in surveillance and partially alleviate the need to generate high-quality databases prior to commencing a surveillance program.

Rather than metabarcoding replacing the role of traditional diagnostics, this study highlights the importance of maintaining taxonomic expertise that can follow up detections and place the results of high-throughput methods in a broader systematic context. In fact, availability of taxonomic expertise may remain a limiting factor for surveillance, as in this study confirming detections took significantly longer than any other part of the metabarcoding pipeline. Conventional DNA barcoding and morphological taxonomy currently benefit from a close and reciprocal interaction, and we believe that integration of non-destructive DNA extraction into metabarcoding protocols will lay the foundation of a robust quality assurance framework for high-throughput insect surveillance.

## Supplementary Information

Supplementary Information.

## Data Availability

The COI, 12S, and 18S reference sequences generated for the aphid and psyllid species used in the mock communities have been uploaded to GenBank (accessions MW804274-MW804279; MW804905-MW804916). The COI sequence generated for the aphid species detected in the trap sample has also been uploaded to GenBank (accession MW804280). The unprocessed FASTQ files generated by the MiSeq have been uploaded to the NCBI Sequence Read Archive (Project ID PRJNA716058). The curated database used to assign taxonomic classification to the sequencing reads and the combined ASV table generated are available at 10.5281/zenodo.3557020. All scripts used to produce the reference database and perform the metabarcoding analyses are available from https://github.com/alexpiper/HemipteraMetabarcodingMS.
