# Supplementary material for: Developing a non-destructive metabarcoding protocol for detection of pest insects in bulk trap catches
Source: Sci Rep. 2021 Apr 12;11:7946. doi: 10.1038/s41598-021-85855-6 (PMC8041782; doi:10.1038/s41598-021-85855-6)
Supplement: Supplementary file 1 — Supplementary Information. [file 41598_2021_85855_MOESM1_ESM.docx]

# Supplementary Information

**Developing a non-destructive metabarcoding protocol for detection of pest insects in bulk trap catches**

Jana Batovska^1,2*^, Alexander M. Piper ^1,2*^, Isabel Valenzuela^1^, John Paul Cunningham^1,2^ & Mark J. Blacket^1^

^1^ Agriculture Victoria, AgriBio, Centre for AgriBioscience, 5 Ring Road, Bundoora, Victoria, 3083, Australia.

^2^ School of Applied Systems Biology, La Trobe University, Bundoora, Victoria, 3086, Australia.

^*^ These authors contributed equally to this work.

**Table S1:** Percentage of each species present in the different sized mock communities based on number of individuals.

|  | **100 Pools** | | | | | **250 Pools** | | | | | **500 Pools** | | | | | **1000 Pools** | | | | |
| --- | --- | --- | --- | --- | --- | --- | --- | --- | --- | --- | --- | --- | --- | --- | --- | --- | --- | --- | --- | --- |
|  | **1** | **2** | **3** | **4** | **5** | **1** | **2** | **3** | **4** | **5** | **1** | **2** | **3** | **4** | **5** | **1** | **2** | **3** | **4** | **5** |
| ***Acizzia***  ***alternata*** | 94.0 | 0.0 | 15.0 | 34.0 | 25.0 | 94.0 | 0.0 | 16.0 | 34.4 | 24.8 | 94.8 | 0.0 | 15.8 | 35.8 | 25.0 | 94.9 | 0.0 | 15.9 | 35.9 | 25.0 |
| ***Acizzia***  ***solanicola*** | 5.0 | 0.0 | 30.0 | 15.0 | 25.0 | 5.6 | 0.0 | 31.6 | 15.6 | 25.2 | 5.0 | 0.0 | 31.4 | 15.8 | 25.0 | 5.0 | 0.0 | 31.7 | 15.9 | 25.0 |
| ***Bactericera cockerelli*** | 1.0 | 0.0 | 5.0 | 1.0 | 0.0 | 0.4 | 0.0 | 2.0 | 0.4 | 0.0 | 0.2 | 0.0 | 1.0 | 0.2 | 0.0 | 0.1 | 0.0 | 0.5 | 0.1 | 0.0 |
| ***Diuraphis***  ***noxia*** | 0.0 | 1.0 | 1.0 | 5.0 | 0.0 | 0.0 | 0.4 | 0.4 | 2.0 | 0.0 | 0.0 | 0.2 | 0.2 | 1.0 | 0.0 | 0.0 | 0.1 | 0.1 | 0.5 | 0.0 |
| ***Metopolophium dirhodum*** | 0.0 | 5.0 | 15.0 | 30.0 | 25.0 | 0.0 | 5.6 | 15.6 | 31.6 | 25.2 | 0.0 | 5.0 | 15.8 | 31.4 | 25.0 | 0.0 | 5.0 | 15.9 | 31.7 | 25.0 |
| ***Rhopalosiphum padi*** | 0.0 | 94.0 | 34.0 | 15.0 | 25.0 | 0.0 | 94.0 | 34.4 | 16.0 | 24.8 | 0.0 | 94.8 | 35.8 | 15.8 | 25.0 | 0.0 | 94.9 | 35.9 | 15.9 | 25.0 |

**Table S2:** Primers used to generate reference DNA sequences for aphids and psyllids used in the mock communities. COI = *Cytochrome oxidase I*, 12S = 12S ribosomal RNA (rRNA), 18S = 18S rRNA. PCR conditions: annealing temperature 50^o^C, x 40 cycles (for all three loci). The GenBank accession numbers are listed for the reference sequences generated using these primers.

| **Primer names** | **Locus** | **Primer sequences (5’ – 3’)** | **Reference** | **Taxa** | **Size (bp)** | **GenBank accessions** |
| --- | --- | --- | --- | --- | --- | --- |
| LCO1490  HCO2198 | COI | GGTCAACAAATCATAAAGATATTGG  TAAACTTCAGGGTGACCAAAAAATCA | Folmer *et al.* 1994 | Aphids | 709 | MW804277 - MW804279 |
| PsyllidCOI-F *  HCO2198 | COI | ACAATTGTTACWGCWCAYGC  TAAACTTCAGGGTGACCAAAAAATCA | This study  Folmer *et al.* 1994 | Psyllids ** | 566 | MW804274 - MW804276 |
| Sternorrhyncha12S-F  Sternorrhyncha12S-R | 12S | YTTTAGGYAAGARTGACGGG  CCWATTWTGTGCCAGCMGTTGC | This study | Aphids / psyllids | 551 | MW804905 - MW804910 |
| 18S-F  18S-Rmod | 18S | CTGGTTGATCCTGCCAGAGT  ACCAGACTTGCCCTCCAAT | Ouvrard *et al.* 2000  Martoni, 2017 | Aphids / psyllids | 614 | MW804911 - MW804916 |

* This new primer is located 118 bp into the LCO1490 / HCO2198 5’ COI region.

** Note, *Acizzia solanicola* reference sequences were generated using the new metabarcoding primers SternoCOI-F1 / SternoCOI-R1 (see Table S3).

**Table S3:** The COI, 12S, and 18S metabarcoding primers designed for the aphid and psyllid species used in the mock communities and field traps. The Illumina adapter sequences attached to the primer are highlighted in yellow. Amplicon size combines the target locus, primer, and adapter sequences.

| **Primer name** | **Primer sequence (5' - 3')** | **Amplicon size (bp)** | **Locus size (bp)** |
| --- | --- | --- | --- |
| SternoCOI_F | ACACTCTTTCCCTACACGACGCTCTTCCGATCTATTGGWGGWTTYGGAAAYTG | 337 | 232 |
| SternoCOI_R | GACTGGAGTTCAGACGTGTGCTCTTCCGATCTATRAARTTRATWGCTCCTA |  |  |
| Sterno12S_F | ACACTCTTTCCCTACACGACGCTCTTCCGATCTCAYCTTGACYTAACAT | 354 | 250 |
| Sterno12S_R | GACTGGAGTTCAGACGTGTGCTCTTCCGATCTAAAYYAGGATTAGATACCC |  |  |
| Sterno18S_F | ACACTCTTTCCCTACACGACGCTCTTCCGATCTATGCATGTCTCAGTGCAAG | 398 | 294 |
| Sterno18S_R | GACTGGAGTTCAGACGTGTGCTCTTCCGATCTCGACAGTTGATAAGGCAGAC |  |  |

**Table S4:** The observed relative abundance of each species in each mock community pool based on all three loci (COI, 12S, and 18S). *Accizia alternata* and *A. solanicola* are aggregated for display purposes as these species could not be differentiated by the 18S loci. False positive abundances are highlighted in yellow and false negative in blue, based on a threshold of 0.01%, with NA representing abundances below this rate. Asterisks (*) indicate pools that had all species correctly identified.

|  | ***Acizzia alternata/ solanicola*** | ***Bactericera cockerelli*** | ***Diuraphis noxia*** | ***Metopolophium dirhodum*** | ***Rhopalosiphum padi*** |
| --- | --- | --- | --- | --- | --- |
| **100 Pool 1*** | 99.62% | 0.37% | NA | NA | NA |
| **100 Pool 2*** | NA | NA | 0.43% | 3.63% | 94.97% |
| **100 Pool 3*** | 34.89% | 16.39% | 0.55% | 6.03% | 41.26% |
| **100 Pool 4*** | 72.10% | 0.35% | 2.01% | 10.69% | 13.20% |
| **100 Pool 5*** | 63.69% | NA | NA | 21.46% | 11.68% |
| **250 Pool 1*** | 99.86% | 0.13% | NA | NA | NA |
| **250 Pool 2*** | NA | NA | 0.19% | 3.93% | 95.45% |
| **250 Pool 3*** | 77.75% | 8.98% | 0.69% | 2.52% | 9.40% |
| **250 Pool 4** | 80.49% | NA | 0.59% | 10.97% | 6.57% |
| **250 Pool 5*** | 59.15% | NA | NA | 28.58% | 10.43% |
| **500 Pool 1*** | 99.77% | 0.22% | NA | NA | NA |
| **500 Pool 2*** | NA | NA | 0.17% | 2.64% | 97.01% |
| **500 Pool 3** | 47.47% | 0.55% | NA | 6.88% | 44.61% |
| **500 Pool 4*** | 80.57% | 0.04% | 0.26% | 8.86% | 9.24% |
| **500 Pool 5*** | 51.00% | NA | NA | 15.80% | 32.04% |
| **1000 Pool 1** | 99.86% | NA | 0.13% | NA | NA |
| **1000 Pool 2*** | NA | NA | 0.04% | 1.39% | 98.54% |
| **1000 Pool 3** | 64% | 1.01% | NA | 6.87% | 27.41% |
| **1000 Pool 4*** | 67.03% | 0.50% | 0.12% | 16.98% | 14.02% |
| **1000 Pool 5*** | 73.97% | NA | NA | 9.61% | 15.50% |

**Table S5:** Proportion of reads from each target loci compared to the proportion of aphids and psyllids in the mock community pools. Loci that were present in less than 15% of the total reads are highlighted yellow.

|  | **COI** | **18S** | **12S** | **Proportion psyllids / aphids** |
| --- | --- | --- | --- | --- |
| **100 Pool 1** | 23.87% | 45.21% | 30.88% | Psyllids only |
| **100 Pool 2** | 62.65% | 31.26% | 6.06% | Aphids only |
| **100 Pool 3** | 29.46% | 53.43% | 17.09% | Half psyllids / aphids |
| **100 Pool 4** | 19.95% | 46.72% | 33.31% | Half psyllids / aphids |
| **100 Pool 5** | 25.82% | 50.41% | 23.69% | Half psyllids / aphids |
| **250 Pool 1** | 11.39% | 1.19% | 87.39% | Psyllids only |
| **250 Pool 2** | 87.07% | 2.59% | 10.31% | Aphids only |
| **250 Pool 3** | 25.06% | 3.31% | 71.59% | Half psyllids / aphids |
| **250 Pool 4** | 24.47% | 2.26% | 73.26% | Half psyllids / aphids |
| **250 Pool 5** | 38.49% | 2.37% | 59.10% | Half psyllids / aphids |
| **500 Pool 1** | 8.07% | 27.96% | 63.95% | Psyllids only |
| **500 Pool 2** | 68.13% | 27.79% | 4.04% | Aphids only |
| **500 Pool 3** | 41.38% | 30.79% | 27.79% | Half psyllids / aphids |
| **500 Pool 4** | 26.53% | 32.07% | 41.39% | Half psyllids / aphids |
| **500 Pool 5** | 41.71% | 25.66% | 32.60% | Half psyllids / aphids |
| **1000 Pool 1** | 7.96% | 35.62% | 56.38% | Psyllids only |
| **1000 Pool 2** | 58.63% | 37.29% | 4.06% | Aphids only |
| **1000 Pool 3** | 28.28% | 39.61% | 32.06% | Half psyllids / aphids |
| **1000 Pool 4** | 25.91% | 30.02% | 43.99% | Half psyllids / aphids |
| **1000 Pool 5** | 24.79% | 34.12% | 41.07% | Half psyllids / aphids |

**Figure S1:** Heat map displaying the number of reads demultiplexed in all possible combinations, taking into account switching of either i5 and i7 indexes, with the yellow boxes indicating the actual index combinations used for each sample. The rate of contamination was calculated at 1.08%. The unprocessed reads were taken from the 100, 500, and 1000 pools MiSeq run.


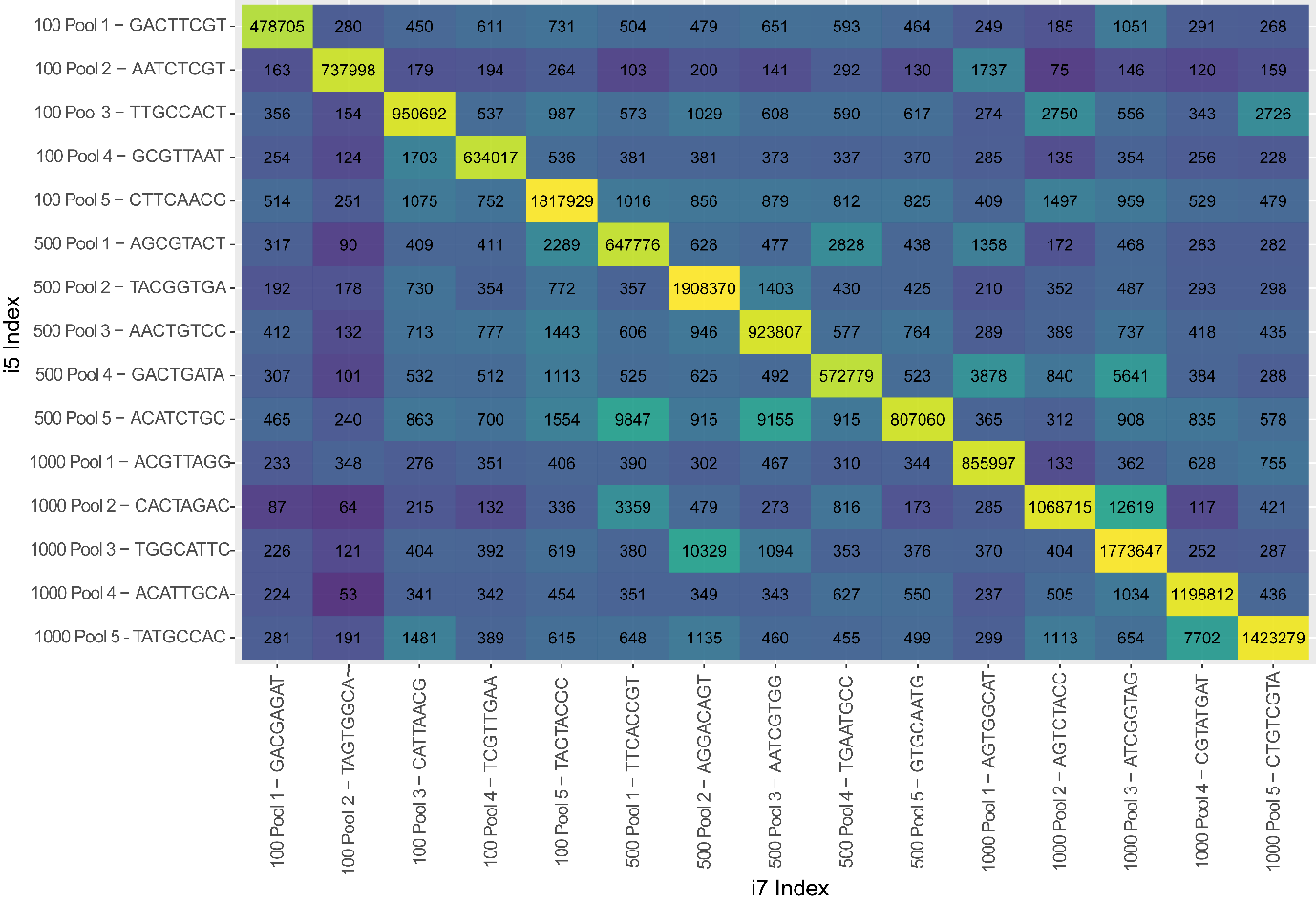


**Figure S2:** Heatmaps displaying the edit distance (including substitutions, insertions and deletions) between combinations for **A** i5 and **B** i7 indices applied in 100, 500, and 1000 pools MiSeq run. Relationship between number of reads assigned to an invalid index combination and the edit distance from the closest index for **C** i5 and **D** i7 indices.


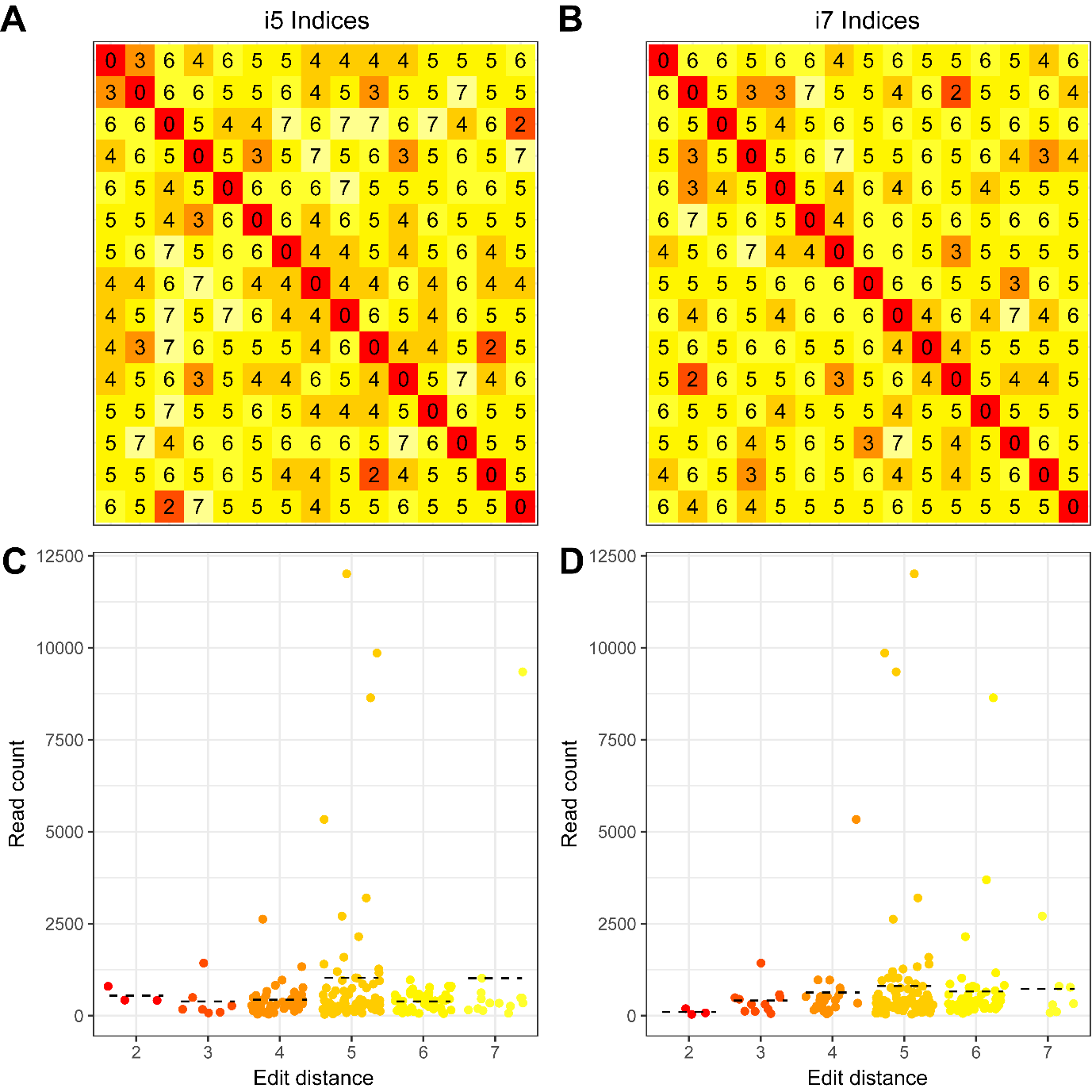


**Figure S3**: A neighbour-joining tree showing the 100% match between COI barcode sequences produced by metabarcoding of Trap 10 and confirmatory barcoding of an aphid abdomen specimen from Trap 10 (highlighted in bold) to an *Aphis varians* COI barcode. Other closely related *Aphis* species are also included, along with their geographical origin and associated BOLD accession numbers. The tree is based on p-distances calculated from a 233 bp alignment. Bootstrap values (%) based on 1,000 replicates are shown next to the branches.


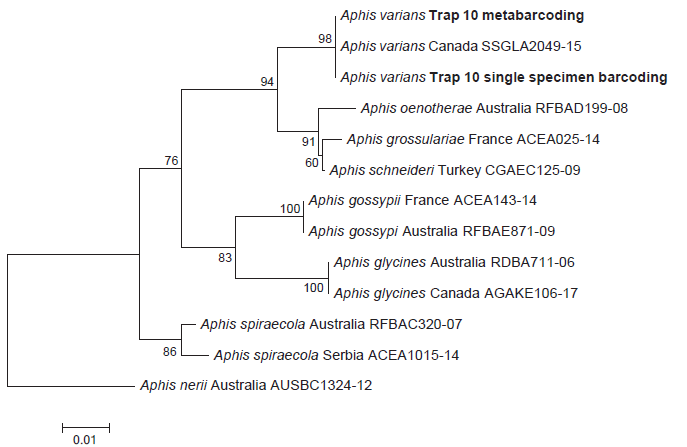


**Figure S4:** Gel images of multiplex PCR for all mock community samples, labelled with the pool sizes and 3 loci. **A)** The 100, 500, and 1000 pool samples. **B)** The separately run 250 pool samples (duplicated to investigate combinatorial and unique dual indexing) with poor 18S amplification.

**A)**
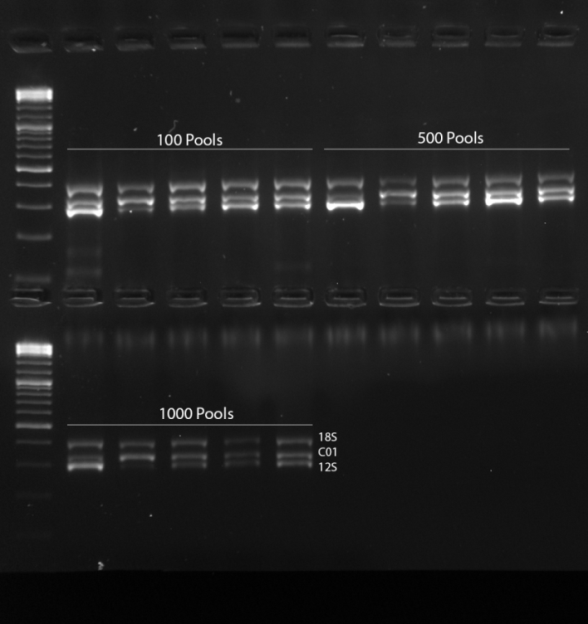


**B)**


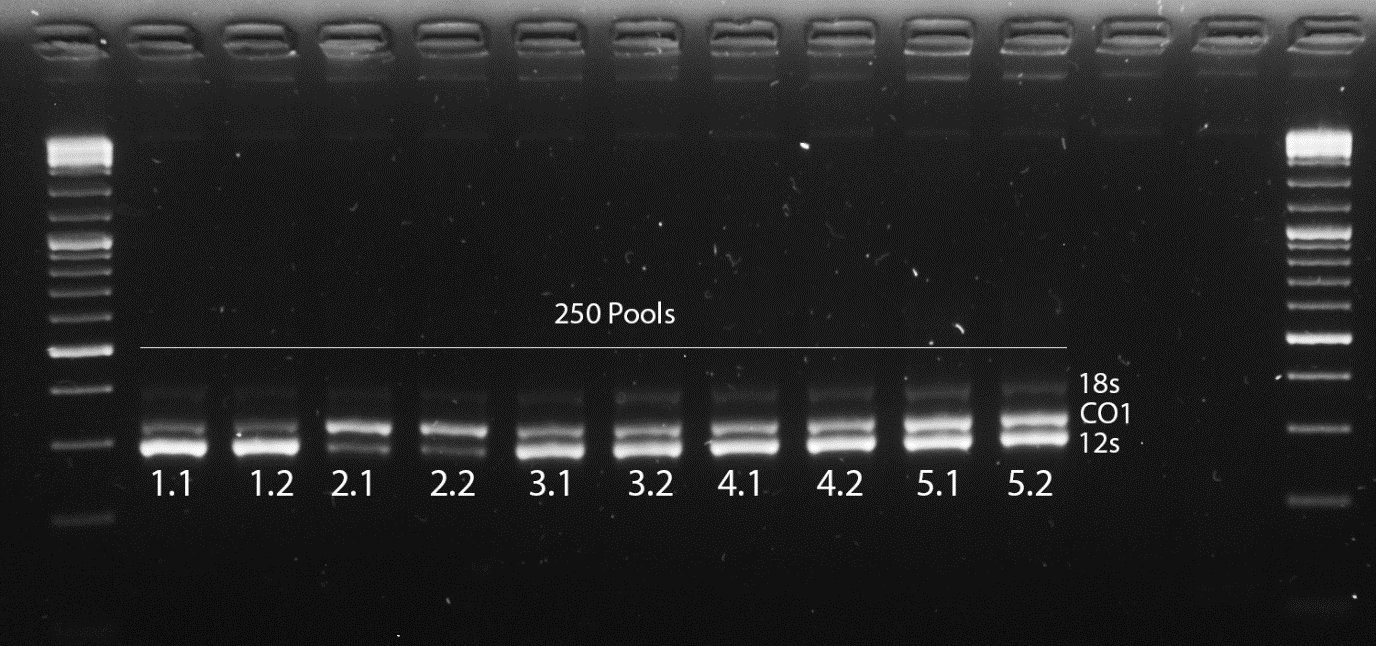


**Appendix S1**

List of filter terms indicating insufficient identifications or common contaminants used to create curated training dataset of reference barcode sequences:

“sp.”, “aff”, "nr.", “cf.”, “nom.”, “nud.”, “environment”, “undescribed”, “unverified”, “uncultured”, “unidentif”, “Bacterium”, “Wolbachia”, “symbiont”, “NA”, “error”.
